# Microbial source tracking identifies sources of contamination for a river flowing into the Yellow Sea

**DOI:** 10.3389/fmicb.2023.1111297

**Published:** 2023-04-24

**Authors:** Jie Ma, Qiuying Lai, Fei He, Xuhan Zhang, Jian Shui, Minghui Yu, Geng Wei, Weixin Li

**Affiliations:** ^1^Nanjing Institute of Environment Sciences, Ministry of Ecology and Environment, Nanjing, China; ^2^College of Hydrology and Water Resources, Hohai University, Nanjing, China

**Keywords:** river, microbial community, the Source Tracker, eutrophication, pollution sources

## Abstract

The excessive input of nutrients into rivers can lead to contamination and eutrophication, which poses a threat to the health of aquatic ecosystems. It is crucial to identify the sources of contaminants to develop effective management plans for eutrophication. However, traditional methods for identifying pollution sources have been insufficient, making it difficult to manage river health effectively. High-throughput sequencing offers a novel method for microbial community source tracking, which can help identify dominant pollution sources in rivers. The Wanggang River was selected for study, as it has suffered accelerated eutrophication due to considerable nutrient input from riparian pollutants. The present study identified the dominant microbial communities in the Wanggang River basin, including Proteobacteria, Actinobacteria, Bacteroidetes, Cyanobacteria, Verrucomicrobia, and Firmicutes. The Source Tracker machine-learning classification system was used to create source-specific microbial community fingerprints to determine the primary sources of contaminants in the basin, with agricultural fertilizer being identified as the main pollutant source. By identifying the microbial communities of potential pollution sources, the study determined the contributing pollutant sources in several major sections of the Wanggang River, including industry, urban land, pond culture, and livestock land. These findings can be used to improve the identification of pollution sources in specific environments and develop effective pollution management plans for polluted river water.

## 1. Introduction

In recent decades, the issue of urban river pollution has become increasingly prevalent due to swift social, industrial, and commercial development (Sinha et al., 2017). In some regions, insufficient sewage treatment facilities have led to the direct release of untreated household waste, livestock and poultry breeding wastewater, and farmland runoff into rivers, causing severe harm to the water quality of the basin (Bu et al., 2011; Milner et al., 2013). Waterways in densely populated and economically developed areas often exhibit eutrophication, excessive heavy metals, dark coloration, unpleasant odor, and other undesirable conditions caused by various pollutants (Williams et al., 2010; Li et al., 2020; Ma et al., 2020). These adverse effects pose a serious risk to regional ecosystems, human wellbeing, and sustainable development (Xu et al., 2014, 2018). To address these challenges and improve the riverine ecological environment, it is essential to identify the distribution patterns and sources of pollution. This will facilitate the development of effective pollution management plans and enable the implementation of efficient river system governance.

Microorganisms are a critical component of the ecological environment, and their dominant species and population diversity vary across different environments, particularly under pollution stress (Staley et al., 2012; Wang et al., 2020; Zemskaya et al., 2021). With the advent of high-throughput sequencing, microbial tracking technology has evolved from being a fecal bacterial indicator to identifying microbial population diversity (Meays et al., 2004; Boehm et al., 2013; Bauza et al., 2019). Microbial high-throughput sequencing provides higher resolution information than traditional source tracking technology, allowing for the establishment of unique microbial fingerprints for both water pollutant sources and monitoring water samples. SourceTracker, a representative microbial source tracking method, uses the Bayesian method to allocate the detected sample sequence to different source environments (Knights et al., 2011). Staley et al. (2018) used the double-blind method to test mixed samples from different pollution sources and verified the accuracy of SourceTracker, which correctly identified 31 pollution sources out of 34 mixtures. Other studies have tentatively identified the main source of phosphorus in Dongting Lake using SourceTracker, based on the significant relationships between the lake sediments, the phosphorus concentration, and the microbial community (Zhang et al., 2019; Gu et al., 2020). The microbial tracking method solves issues of low sample discrimination, single tracking markers, and the inability to accurately distinguish pollution sources within a large watershed that arise with traditional source tracking methods (Kircher and Kelso, 2010; Zhang et al., 2019). While the SourceTracker method has been widely used to track the source of fecal bacteria, antibiotic resistance genes, and sediments, it has been relatively underused for identifying pollution sources in the river and lake systems (Ibarbalz et al., 2013; Zhang et al., 2021).

This study aimed to use SourceTracker to identify the sources of microbial diversity in the Wanggang River by analyzing the contributions of industry, urban land, pond culture, and livestock lands. We combined data from the Bayesian mass balance model for microbial detection with the characteristics and distribution of pollution sources to improve the accuracy of pollution source tracing. The Wanggang River, located in eastern Jiangsu province, has a dense network of water connecting to the sea and suffers from severe eutrophication due to different land uses on both banks. The findings will help to develop more targeted and efficient pollution control strategies not only for the Wanggang River but also for other rivers and sub-basins in the future.

## 2. Materials and methods

### 2.1. Sampling locations

**Figure 1:**
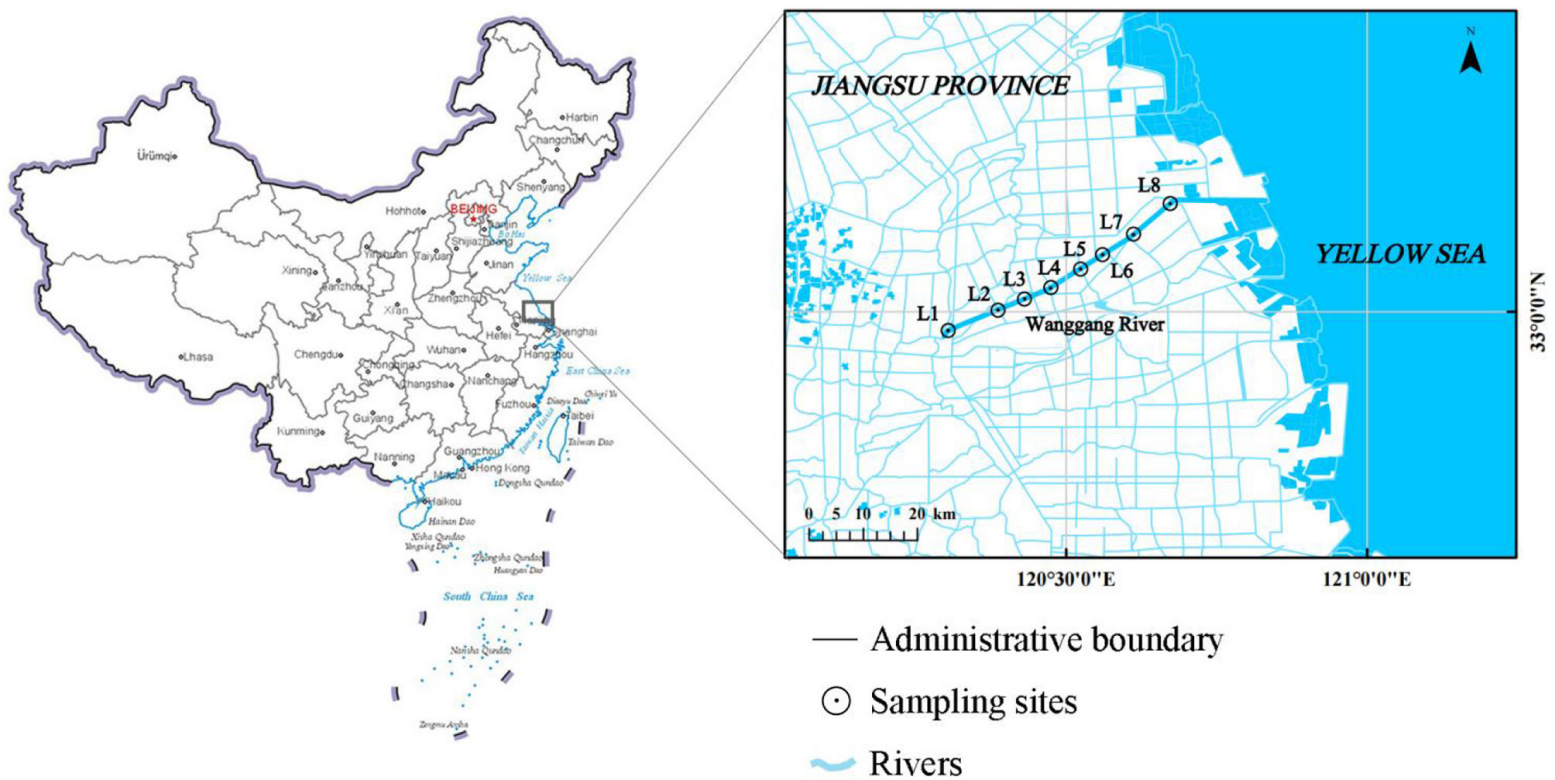
Sampling locations in the Wanggang River.

A total of eight sample sites were selected according to landform characteristics, hydrographic laws, and the type of pollution in the Wanggang River. The river was divided into upstream (L1–L4) and downstream (L5–L8) sections, with the boundary set at relatively densely populated towns. The sampling was conducted in May 2021, during clear weather and with no precipitation in the study area for 7 days. The distribution of sampling is presented in Figure 1. Water samples were collected in triplicate from just below the surface using a plexiglass water sampler and then homogenized. A total of five types of potential pollution sources were sampled in the Wanggang River basin, including livestock land (BS), ponds used for culture (AS), industry (FS), farmland (WS), and urban land (DS). We randomly collected and mixed multiple samples of each potential pollution source from more than three points along the river. Each sample was stored in a 1 L brown wide-mouth bottle, transported on ice to the laboratory, and kept at 4°C for further analysis.

### 2.2. DNA extraction and sequencing analysis

To obtain DNA, 500 ml of samples were filtered onto separate 0.22 mm cellulose acetate filters. The FastDNA Spin kit was used to extract DNA from the samples, following the manufacturer's instructions. The extracted DNA was subsequently submitted for PCR, amplifying the V3–V4 region of 16S rRNA genes using specific primer pairs 338F (5′-ACTCCTACGGGAGGCAGCAG-3′) and 806R (5′-GGACTACHVGGGTWTCTAAT-3′), and then sequenced on the Illumina MiSeq PE250 platform (Illumina, San Diego, CA). After data processing and quality filtering, the UCLUST algorithm was used to assign operational taxonomic units (OTUs) based on 97% identification and compared with the Silva v128 reference database using the PyNAST alignment algorithm. The 16S rRNA gene copy number was normalized using a normalized OTU table. Taxonomic assignments were conducted against the RDP Classifier v2.2 with an 80% threshold.

### 2.3. Statistical analysis

Statistical analyses were conducted using QIIME v1.9.0.40, SPSS v23.0, and R Studio. Alpha diversity was determined using the Chao1 index, the Shannon diversity index, and the Simpson diversity index based on the OTU analysis, with Chao1 measuring species richness and the Simpson and Shannon indices measuring diversity. The Bray–Curtis dissimilarity matrix was used to calculate non-metric multidimensional scaling (NMDS) and explore and visualize the variability of the microbial community structure.

### 2.4. SourceTracker analysis

SourceTracker is a Bayesian algorithm that uses Gibbs sampling to calculate a joint probability distribution based on the microbial community structure in samples as a variable, without relying on specific indicator bacteria as a traceable target (Staley et al., 2018). The R script setting of SourceTracker was used to analyze the source of a contaminating microbial community and establish a source library of potential contamination (industry, urban land, pond culture, livestock land, and farmland) along with their respective microbial communities. The sampling locations L1–L8 were considered pollution sink sites, while FS, DS, AS, BS, and WS were considered pollution source sites for the Wanggang River.

The OTU tables were obtained through quality filtering, and selected OTUs were used as input files. SourceTracker analysis was conducted using default settings with a rarefaction depth of 1,000, burn-in (100), restart (10), alpha (0.001), and beta (0.01), as this has been previously demonstrated to yield high sensitivity, specificity, accuracy, and precision (Henry et al., 2016). For each source, the data were subjected to five independent operations using quadratic calculation methods, and the results were averaged to prevent potential false positive predictions. The relative standard deviation (RSD) was then calculated by dividing the mean predicted proportion by the standard deviation, providing an estimate of the confidence in the mean predicted source proportion and the variance between different models (Zhang et al., 2019).

## 3. Results and discussion

### 3.1. Variance in a river microbial community

**Figure 2:**
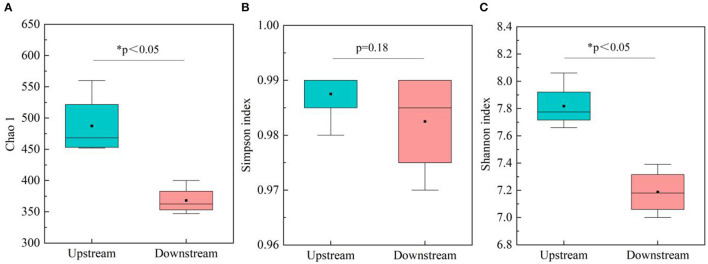
Microbial diversity in upstream and downstream locations (*n* = 24), **(A)** Chao 1 index; **(B)** Simpson index; and **(C)** Shannon index.

The study investigated the variation in microbial diversity by analyzing differences in diversity indices between upstream and downstream locations in the Wanggang River (Figure 2). The results indicated that microbial community richness (Chao 1) and diversity (Shannon index and Simpson index) were higher in the upstream water bodies than in the downstream locations. A *t*-test analysis revealed significant differences (P < 0.05) in Chao 1 between upstream and downstream locations. These findings suggest that microbial communities in the upstream locations are richer and more diverse than those in the downstream areas, possibly due to greater pollution levels in the upstream source, which may harbor a larger variety of microorganisms. The decrease in diversity indices from upstream to downstream could be attributed to the migration and transformation of microorganisms within the river ecosystem (Gu et al., 2020).

**Figure 3:**
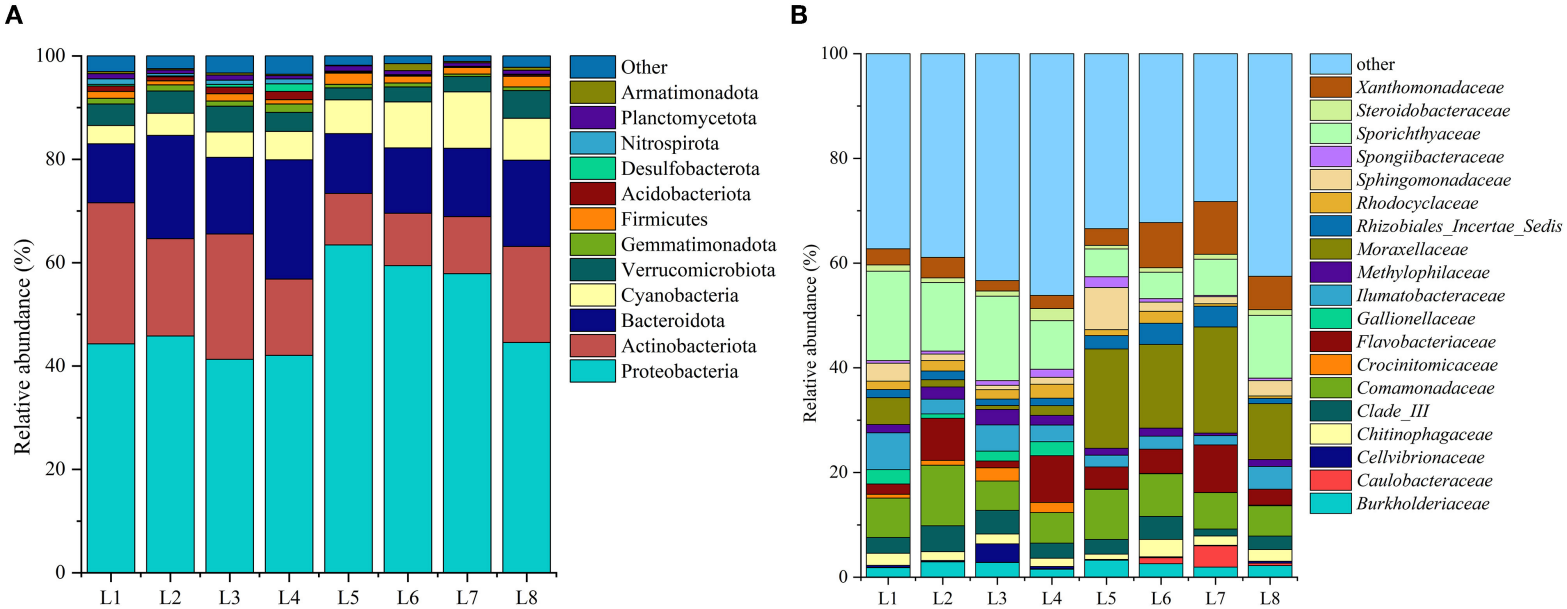
The microbial community structure, **(A)** at the phylum level, **(B)** at the genus level.

Among all samples, Proteobacteria was the most prevalent phylum in all samples, accounting for 41.30–63.64% of the bacterial population (Figure 3A). Proteobacteria play a variety of biogeochemical processes in lakes and other aquatic ecosystems (Zhang et al., 2015). In the Wanggang River, the most common types were γ-Proteobacteria (average 37.53%) and α-Proteobacteria (average 12.30%). Other dominant phyla included Actinobacteria (average 16.85%), Bacteroidetes (average 15.43%), Cyanobacteria (average 6.60%), Verrucomicrobia (average 3.82%), and Firmicutes (average 1.40%). Bacteroidetes are crucial to the breakdown of complex molecules in fresh water, including cellulose and chitin (Ma et al., 2021). In addition, certain dominant phyla (accounting for >1% of the population) were prevalent at specific sites, such as Acidobacteria observed at L1 and L3; Gemmatimonadetes at L1, L2, and L4; and Planctomycetes at L1, L3, and L5, a phylum strongly associated with total nitrogen content (Hempel et al., 2008). The Kruskal–Wallis test analysis showed that the abundance of Proteobacteria, Acidobacteriota, Cyanobacteria, Gemmatimonadota, and other bacteria was significantly higher in the upstream water bodies than in the downstream water bodies (P < 0.05), which was comparable with the microbial diversity index evaluation. Furthermore, only 2.3% of the sequences from the eight sample sites were unable to be classified at the phylum level and were therefore designated as “unclassified bacteria.” These findings demonstrate the high accuracy of our high-throughput sequencing, as the vast majority of sequence results were successfully classified.

At the family level, 232 families were identified in the bacterial communities from the eight sample sites, with 46 of them having a relative abundance >1% (Figure 3B). Of these, four dominant families, with an average abundance of more than 5%, were identified as Sporichthyaceae (average 10.62%), Moraxellaceae (average 9.38%), Comamonadaceae (average 7.62%), and Flavobacteriaceae (average 5.16%). Moraxellaceae is a gram-negative, non-fermentative, aerobic, or facultative anaerobic bacterium. It is worth noting that the abundance of Moraxellaceae was significantly higher in downstream locations, which may be attributed to the discharge of wastewater from sites of pond culture that are distributed downstream.

### 3.2. Potential pollution sources and their microbial characteristics

**Figure 4:**
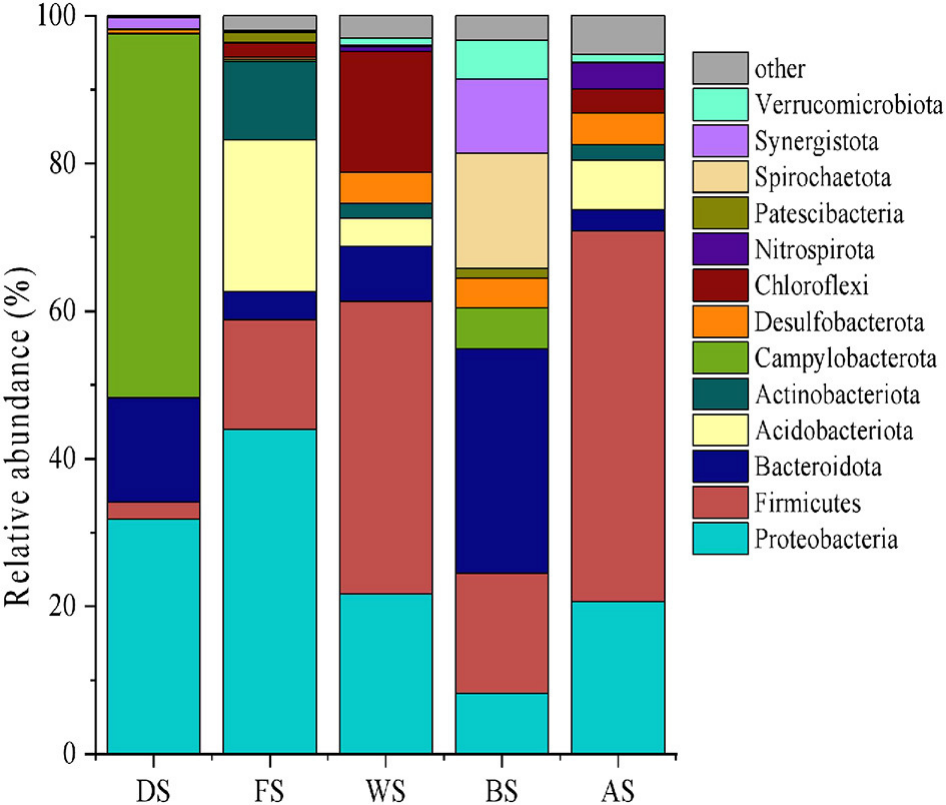
The microbial community structure at the phylum level for potential pollution sources (FS, industry; DS, urban land; AS, culture ponds; BS, livestock; WS, farmland).

The pollution source samples were analyzed through high-throughput sequencing and biological information analysis at the phylum level, and dominant strains with average abundance (>1%) were selected (Figure 4). By examining the microbial diversity of the different pollution sources, the sources were preliminarily characterized. For instance, livestock wastewater had a higher abundance of Bacteroidetes than Firmicutes and Spirochaetes. Campylobacterota and Proteobacteria were enriched in domestic sewage. Previous studies have shown the seasonal variation of *Campylobacter* in sewage (Jones et al., 1990). Farmland wastewater had a wide presence of Proteobacteria, Firmicutes, and Chloroflexi, a group of bacteria that produce energy through photosynthesis (Chang et al., 2021).

The study conducted by Hartmann et al. (2014) also focused on tracking shifts in soil and water microbial communities after a simulated manure application, and their findings were comparable to ours. We found that Proteobacteria and Actinobacteria were the dominant bacteria in industrial sources of pollutants. Alphaproteobacteria were widely found in industrial wastewater treatment plants and were involved in the degradation of halogenated benzoic acid, a key intermediate in dye production (Caroline et al., 2005). Actinobacteria were heterotrophic bacteria that thrived in organic-rich water bodies (Zhang et al., 2015). Finally, our study revealed that Proteobacteria and Firmicutes were the most abundant bacteria in samples collected from pond culture. Although previous studies have found antibiotic residues in aquaculture wastewater, the abundance of antibiotic-resistant Planctomycetes was low in our study.

**Figure 5:**
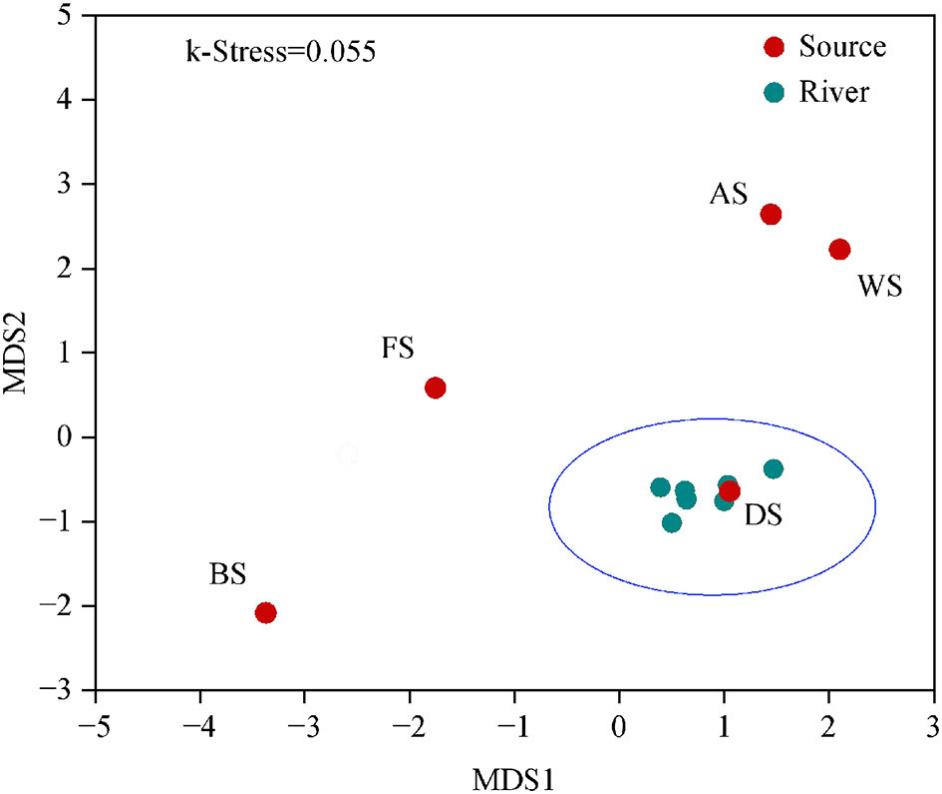
The non-metric multidimensional scaling analysis (NMDS) of bacterial community composition based on the Bray–Curtis dissimilarity matrix (FS, industry; DS, urban land; AS, culture ponds; BS, livestock; WS, farmland).

**Figure 6:**
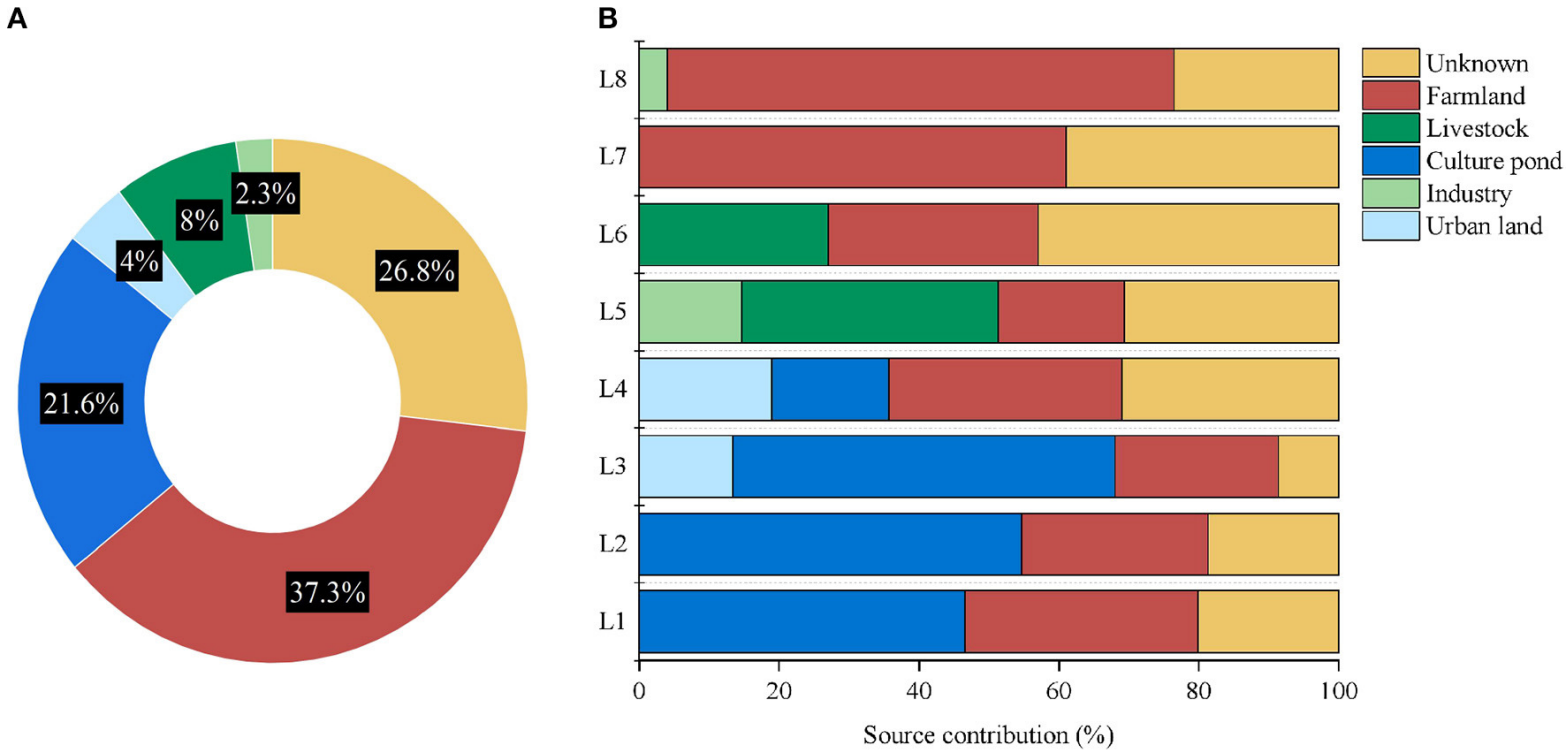
Contribution of pollution sources, **(A)** Average contribution of different pollution sources to the Wanggang River and **(B)** the contribution of different pollution sources at each sampling location.

By utilizing non-metric multidimensional scaling analysis and similarity analysis based on the Bray–Curtis dissimilarity matrix, the beta diversity of bacterial communities in the Wanggang River was compared. The microbial communities from the pollution source were significantly different and highly dispersed (Figure 5), while those from the river samples were clustered together. This result is a consequence of the natural environment and human activity. Pollution sources determine microbial communities, but in natural systems, such as rivers, hydrodynamics constantly homogenize the communities. As a result, the river water samples are clustered together. The observed significant differences in microbial community structure and diversity among source samples support the feasibility and accuracy of the SourceTracker model for pollutant tracing (Figure 6).

### 3.3. Main sources of water pollution in the Wanggang River

The contribution of the sources in several major regions of the Wanggang River was determined by identifying the microbial communities of potential pollution sources. The average contribution of pollution sources to each river sampling site of the Wanggang River is shown in Figure 6A. In general, farmland water has the greatest impact on the Wanggang River basin, accounting for 37.3%, followed by pond culture water accounting for 21.6%. The contribution of urban domestic sewage, livestock, and industrial pollution is <10% to the Wanggang River pollution. Aquaculture contributed significantly to water pollution in the upstream location, with the average contribution in the four sites in the upper region reaching 43%. We also found only a minor amount of pollution from domestic sewage only at L3 and L4 in the upper regions. In the downstream, a small amount of industrial pollution was observed at L5 and L8. At L5 and L6, the contribution of pollution from livestock lands was found to account for 30%, while the contribution of farmland wastewater in L7 and L8 was identified to be 67%.

Farmland water was the primary contributor to pollution in the Wanggang River, likely due to the significant number of farms situated along the river (Ma et al., 2022). Domestic sewage had a minimal impact on pollution, as it is mostly piped away from the river, resulting in low levels of untreated sewage in natural water bodies. Industrial pollution was also relatively low, although some wastewater discharge from textile factories may affect water quality. In the upstream locations, aquaculture contributed significantly to pollution, possibly due to the distribution of pond culture sites. Unknown sources contributed an estimated 8.67% to 43.00% of the bacterial community, indicating that some of the potential pollutants were not studied or included in this research.

This study employs SourceTracker, a machine learning-based tool, to investigate the distribution of microbial communities in a river system and trace sources of pollutants in the complex source condition. In this study, microbial tracking technology can use host-specific or host-related indicators to identify the sources and contributions of human and non-human pollution sources (Knights et al., 2011; Gu et al., 2020). However, this technology has limitations, including the potential for identification inaccuracies if sample selection is insufficient and the need for further optimization in setting program parameters (Zhang et al., 2019). In addition, some scholars argue that microbial-oriented SourceTracker is limited in its ability to analyze pollution sources beyond domestic sewage and livestock wastewater (Bauza et al., 2019). To comprehensively and accurately identify pollution sources in complex water bodies, combining multiple tracking methods and approaches is a promising research direction for the future. By utilizing the respective advantages of different tracking methods and analyzing sources from multiple perspectives, we can improve source identification accuracy.

## 4. Conclusion

In this study, microbial tracking was utilized to identify the primary pollution sources (industry, urban land, pond culture, livestock land, and farmland)and determine their respective contributions to pollution in the Wanggang River. The study aimed to improve source tracking accuracy in a water system with high pollution levels and complex pollution sources. The results of the study can help river water managers to evaluate water ecology and environmental risks at regional and watershed scales and to develop effective management plans to mitigate pollution and its negative impacts.

## Data availability statement

The original contributions presented in the study are included in the article/supplementary material, further inquiries can be directed to the corresponding author.

## Author contributions

WL contributed to the study's conception and design. QL, JS, GW, MY, and XZ performed material preparation, data collection, and analysis. JM and FH wrote the first draft of the manuscript. All authors contributed to the article and approved the submitted version.
